# Poverty and caregiver mental health are related to infant and toddler resting-state brain networks: insights from fNIRS

**DOI:** 10.1117/1.NPh.13.S1.S13014

**Published:** 2026-08-14

**Authors:** Kaja K. Jasińska, Hannah L. Whitehead, Sharon Wolf, Fabrice Tanoh, Stephanie Bugden, Brooke Wortsman, Amy Ogan

**Affiliations:** a University of Toronto, Toronto, Ontario, Canada; b Haskins Laboratories, New Haven, Connecticut, United States; c University of Pennsylvania, Philadelphia, Pennsylvania, United States; d Peleforo Gon Coulibaly University, Khorogo, Côte d’Ivoire; e University of Winnipeg, Winnipeg, Manitoba, Canada; f Carnegie Mellon University, Pittsburgh, Pennsylvania, United States

**Keywords:** graph theory, poverty, maternal mental health, infants, toddlers, brain development

## Abstract

**Significance:**

The first 3 years of life represent a period of heightened neural plasticity, during which early adversity—such as poverty and maternal stress—can shape long-term developmental trajectories. However, little is known about how these adversities are linked to neural network organization in infants and toddlers living in low-resource settings.

**Aim:**

We examined how prenatal exposure to maternal poverty and mental health symptoms relates to neural network organization in early childhood.

**Approach:**

We conducted a longitudinal study with 42 children (6 to 36 months old) from economically vulnerable households in rural Côte d’Ivoire. Maternal multidimensional poverty and mental health symptoms were assessed around conception and during pregnancy. Two years later, children’s development was measured using the Caregiver Reported Early Development Instruments, and resting-state brain activity was recorded using portable functional near-infrared spectroscopy. Graph-theoretic metrics of functional connectivity, including segregation, integration, and small-worldness, were computed to characterize neural network organization.

**Results:**

Greater exposure to poverty was associated with reduced network segregation and integration. By contrast, higher maternal mental health symptoms were linked to increased segregation and integration. These findings suggest that deprivation and maternal stress differentially relate to early neural network architecture.

**Conclusions:**

Our results align with dimensional models of adversity and suggest that deprivation (related to poverty) and threat/unpredictability (related to maternal mental health and caregiving) may become biologically embedded. These findings underscore the importance of addressing both structural poverty and maternal mental health during pregnancy and early childhood to promote healthy brain development in high-adversity contexts.

## 1 Introduction

Between birth and 5, the developing brain is highly sensitive to the effects of the environment. During this period of peak brain plasticity, or sensitive period,^1^^,^^2^ poverty and its co-occurring risks (e.g., food insecurity, infectious disease, lack of cognitive stimulation, maternal stress, and mental health) can adversely affect neurocognitive development. Compared with other regions, sub-Saharan Africa has the largest number of young children experiencing poverty,^3^ as well as the largest number and proportion of 3- to 4-year-olds (29.4 million; 44%) failing to meet cognitive, language, and social-emotional milestones.^4^ Côte d’Ivoire ranks at the bottom of the region, at 170 of 189 countries in the Human Development Index, a statistic measuring population life expectancy, education, and health.^5^ 28.2% of the population lives below the poverty line,^6^ and in some rural communities, the poverty rate is 61.2%,^7^ with many households surviving on $1 to 2 a day.^8^ In this study, we investigated the relation between maternal mental health and exposure to multiple dimensions of poverty and infant and toddler brain development in rural Côte d’Ivoire using portable functional near-infrared spectroscopy (fNIRS). We leveraged graph theoretic approaches to measure developing neural networks and explored multiple mechanisms by which perinatal environments are prospectively associated with early brain development, specifically, early deprivation (e.g., poverty) and exposure to stress (e.g., poor maternal mental health) in 6- to 36-month-old infants and toddlers.

### 1.1 Poverty and Brain Development

A large body of research has established the negative effects of poverty on physiological and neurobiological development, including neural structure, function, and network organization. These effects are central to the well-documented poverty-related disparities in academic achievement, mental health, and life outcomes. More recently, childhood poverty has also been implicated in overall neural network organization, including functional integration and segregation.^9^ Functional segregation enables specialized and efficient processing within specific brain regions or networks, such as those responsible for sensory and motor functions, by promoting strong local connectivity among nearby neurons.^10^^–^^12^ Brain areas involved in fundamental sensory and motor functions develop early, forming a foundation for more advanced cognitive processing.^13^ By contrast, functional integration supports communication between specialized regions through long-range connections, which becomes increasingly crucial as cognitive tasks grow in complexity and require the coordination of multiple processes, including memory, attention, and executive control.^14^^,^^15^ Childhood poverty has been linked with functional integration in emotion networks^16^ and segregation in whole-brain, somatomotor, ventral attention, and limbic networks.^17^

The negative effects of poverty on brain function and structure are already apparent in infancy. For example, an EEG study of 160 1-month-old infants’ resting-state brain activity showed that lower family income was associated with developmental differences in brain activity.^18^ Specifically, low family income was associated with more lower-frequency (theta) and less higher-frequency (beta and gamma) power. Lower-frequency power decreases and higher-frequency power increases over development are thought to reflect cortical maturation and more efficient neural organization.^19^ Higher-frequency power is linked with higher language, cognitive, and socioemotional outcomes, whereas lower-frequency power is linked with behavioral, attention, learning problems, and increased risk of psychopathology in childhood.^20^^–^^23^

### 1.2 Mechanisms Linking Poverty to Brain Development

Poverty is related to brain development and childhood outcomes through several interconnected mechanisms. According to the Dimensional Model of Adversity and Psychopathology,^24^^,^^25^ poverty is often associated with major threats and deprivations that impact healthy development. These risk factors include poor nutrition, poor maternal mental health, and subsequently, unpredictable caregiving. Taken together, compounding risks manifest an environment of potential threat, deprivation, and unpredictability during the perinatal stages, when neurodevelopment is at its most vulnerable.

For example, adequate nutrition is essential for early brain development, beginning before conception,^26^ in utero,^27^ and continues to support brain development during early childhood and beyond.^28^^,^^29^ Many aspects of neural development require caloric energy and specific micronutrients; this includes neuronal cell proliferation and differentiation, growth of axons and dendrites, synaptogenesis, myelination, and programmed cell death (a fine-tuning of existing neural connections), as well as the synthesis and activity of neurotransmitters that underlie cognitive processes and mood.^30^ Children who have experienced deprivations in nutrition prenatally and in the first few years of life are more likely to have lower IQ, poorer cognitive abilities, greater behavioral problems (e.g., poor attention control), and poorer educational outcomes,^31^^,^^32^ and these negative consequences can persist for decades.^33^^,^^34^ For example, a study of 179 infants and children in rural Gambia found that iron deficiency at 5 and 12 months old was negatively associated with cognitive development.^35^

Mothers raising children in poverty also face a daunting array of physical and psychosocial threats that undermine their mental health and diminish their capacity for responsive and predictable parenting, limiting the amount and quality of stimulating experiences essential for neurocognitive development, which in turn negatively impacts children’s development.^36^ Poor maternal mental health is associated with the dysregulation of the hypothalamic-pituitary-adrenal axis in infants.^37^ Infants of depressed mothers tend to have greater cortisol reactivity to stress.^38^^–^^40^ Infants who were prenatally exposed to maternal depression showed poorer connectivity within the circuitry connecting the amygdala with the prefrontal cortex^41^ and white matter microstructure.^42^ Furthermore, maternal anxiety has been found to relate to unpredictable caregiving^43^^,^^44^ which thereby impacts cognitive development.^45^ Critically, mothers’ prenatal psychological distress has long-term impacts on behavior and socio-emotional development as well as cognitive and language outcomes of school-aged children.^46^ Furthermore, maternal mental health has been linked to deprivations in their child’s nutritional status,^47^ which may further compound the negative effects on child development associated with both poor maternal mental health and undernutrition. For example, in a study of 468 mother-infant pairs in Brazil, maternal depression and anxiety was found to modify the strength of the relationship between household food insecurity and child development outcomes.^48^

### 1.3 Poverty and Brain Development in the Majority World

Although the global burden of poverty and its effect on child development are greater in low- and middle-income countries across the Majority World, most studies of the association between poverty and neurocognitive development focus on poverty in high-income countries in the Minority World. Of the limited existing work in the Majority World, a study in Bangladesh finds that household wealth is associated with EEG oscillations in 36-month-olds. More specifically, greater poverty is related to greater absolute activation in beta and gamma bands across frontal, central, and parietal brain regions.^49^ Thus, greater poverty may be associated with delayed patterns of EEG power, which has been used as an indicator of brain maturation. The authors conclude that infants may experience delayed neurocognitive development as a function of poverty exposure. Additional work by this team has found relations between malnutrition and neurocognitive development,^50^ as well as relations between pro-inflammatory markers and neurocognitive development.^51^ A functional near-infrared spectroscopy (fNIRS) study compared neural habituation responses of infants 5- and 8-month-olds in a high-income context (UK) and a low-income poverty context (Gambia) using a habituation and novelty detection paradigm.^52^ The paradigm allowed researchers to test whether infants exhibit discriminatory neural responses to auditory stimuli, which are associated with attention, learning, and memory. Gambian infants (exposed to greater poverty) exhibited an attenuated habituation response compared with UK infants.^52^ In other words, Gambian infants, on average, required more trials prior to habituation than infants from the UK. These studies highlight that relations between poverty and development persist in Majority World contexts. More specifically, higher levels of poverty may be associated with delayed development across structural and functional indicators of brain development. That said, our understanding of neurocognitive development in Majority World contexts remains limited to research from a handful of studies and typically focuses on specific developmental processes across critical ages, and concurrent, rather than predictive, associations between poverty and neurocognition. We contribute to expanding the body of literature by investigating the experiences of infants and toddlers growing up in rural communities with high poverty in Côte d’Ivoire. Importantly, rather than capturing neural responses associated with specific cognitive tasks (i.e., novelty detection), here, we examined broad properties of neural network organization.

### 1.4 Measuring Brain Networks

Modeling the brain as a complex network provides a more comprehensive understanding of the functional architecture of the human mind that functional activation maps alone, including functional integration and segregation.^9^ Functional connectivity in infants and toddlers can be measured by examining neural activity across distributed brain regions using fNIRS. Graph-theoretic network analyses can show the intrinsic topological organization of brain networks, inform how the brain supports global and local information transfer,^15^ and capture aspects of the brain’s functional segregation and integration.

Biological networks typically show a “small-world” architecture; meaning, they are neither completely random nor completely regular. Small-world networks show high local efficiency and global efficiency in information transfer, with low wiring costs.^53^^,^^54^ Notably, small-world networks optimize functional segregation and integration because they offer both high global and local information transfer.

At birth, adult-like topological organization of structural brain networks is already established^12^^,^^55^^–^^58^ and continues to mature throughout infancy, childhood, and beyond.^59^^–^^61^ In the first two years of life, the topological organization of structural brain networks shows increased global efficiency (i.e., 2-year-olds compared with 6-month-olds^62^) and global information integration (i.e., decreased average path length in 6-month-olds versus neonates^56^). Regional brain hubs are also well established at birth and observed in the superior parietal lobule, cuneus, operculum, Heschl’s gyrus, and sensorimotor areas.^55^^,^^57^^,^^62^ Similarly, small-world organization is also present at birth, and in the first few years of life, functional network segregation and integration increase.^63^ Over early childhood (i.e., 2 to 8 years), a shift from global information integration to greater local information segregation is observed, reflecting increased regional functional specialization.^64^

### 1.5 Current Study

The present longitudinal study aimed to better understand the link between exposure to poverty and emerging neural networks. We asked, is exposure to poverty and stress associated with poor maternal mental health around the time of conception and in utero related to neural networks in infants and toddlers? To this goal, we measured poverty and maternal mental health in economically vulnerable mothers in rural Côte d’Ivoire. Two years later, we captured measures of infant development and neural network organization and functional connectivity in their infant and toddler children using a parental developmental inventory and fNIRS. fNIRS is a safe and noninvasive neuroimaging method that measures the brain’s hemodynamic response. It is particularly well suited to studies with young children as it tolerates movement well. The portability and ease of fNIRS (i.e., no need for magnetic shielding or specialized facilities) permit mobile neuroimaging studies in understudied and underrepresented groups in low-resource and challenging contexts (i.e., rural communities with limited infrastructure for laboratory testing).^65^^,^^66^ Several advances in resting-state functional connectivity and network organization analytic approaches for fNIRS over the past decade have demonstrated the appropriateness of this neuroimaging technique for network analyses.^67^^–^^69^

Specifically, we measured inter- and intra-hemispheric correlation and key indicators of functional integration (global efficiency, degree centrality, shortest-path length) and segregation (clustering coefficient), and small-worldness during a resting-state scan. We hypothesized that exposure to poverty and stress would be negatively associated with child development and neural network organization. We predicted that infants and toddlers with greater exposure would show reduced functional connectivity and reduced network integration and segregation.

## 2 Materials and Methods

### 2.1 Participants

Sixty-one infants and toddlers ($N_{\text{males}}=33$) between the ages of 6 and 36 months ($M_{\text{age}}=14.1\text{ months}$, ${\mathrm{SD}}_{\text{age}}=6.88$) and their mothers ($M_{\text{age}}=34.1\text{ years}$, ${\mathrm{SD}}_{\text{age}}=7.31$, range=21 to 59 years) participated in the study. Participants lived in rural communities in the Daloa and Bouafle region in central Côte d’Ivoire and were participating in a larger randomized control trial (RCT) examining the impacts of cash transfers and quality education on families and children in cocoa-producing communities.^70^ Families were recruited into the study between March and September 2021 (time 1). Mothers and infants and toddlers participated in the neuroimaging between April 2023 and May 2024 (time 2).

Due to technical difficulties and participant fussiness, nine infants and toddlers did not complete the resting-state fNIRS scan, two infants’ mothers did not complete the Caregiver Reported Early Development Instruments (CREDI) questionnaire (one participant completed neither NIRS or CREDI), and nine participants had low signal quality (defined below in data processing) and were excluded from further analysis. Importantly, the nine participants excluded on the basis of data quality did not differ from the remaining participants in age (b(SE)=−4.350(3.199), t(59)=−1.36, p=0.18, n.s.), poverty score (b(SE)=−0.012(0.076), t(59)=−0.15, p=0.88, n.s.), CREDI development score (b(SE)=0.169(0.551), t(54)=0.31, p=0.76, n.s.), or gender ($\chi^{2(1,58)}=0.03$, p=0.9, n.s.). Forty-two infants ($N_{\text{males}}=20$) between the ages of 6 and 36 months ($M_{\text{age}}=15.1$, ${\mathrm{SD}}_{\text{age}}=7.66$) were retained for neuroimaging analyses. Of the remaining sample (n=42), at time 1, 15 mothers were pregnant, two had a 2-month-old infant, and 25 mothers would become pregnant within the year (mean time to pregnancy = 6.7 months). Of those mothers who were pregnant, seven were in their first trimester, four were in the second trimester, and four were in their third trimester. Of 42 mothers analyzed in this study, 28 were recipients of a cash transfer program; however, an analysis of treatment effect on functional connectivity and neural network organization in infants and toddlers is beyond the scope of the present study due to our limited sample size. The study received ethical approval from the University of Toronto Research Ethics Board.

#### 2.1.1 Inclusion/exclusion criteria

##### 2.1.1.1 Communities

The larger RCT provided cash transfers (∼10 USD/week) for 100 weeks to economically vulnerable mothers in cocoa-producing households (implemented by the Dutch NGO 100WEEKS) and improved education quality in community schools (implemented by the Ivorian Ministry of Education). Community eligibility, therefore, considered cocoa production and education. To reach cocoa-producing families, communities were included in the study if they were affiliated with a cocoa cooperative. 100WEEKS conducted in-person visits to each cooperative, verified their producer registries (as part of 100WEEKS operations), and recruited eight cooperatives as research partners. Cooperatives provided a list of communities and cocoa producers. Communities with a primary school were eligible for the study. Of the 207 eligible communities, 140 were randomly selected, and half were randomized to treatment (cash transfer) and control groups.

##### 2.1.1.2 Families

Fifteen women in each community participated in the larger RCT. Women were included in the RCT if their households produced cocoa and were affiliated with a partner cocoa cooperative, were economically vulnerable (i.e., bottom 20th percentile in their community), and had at least one child between 5 and 15 years of age. Economic vulnerability was determined by 100WEEKS in partnership with each community; 100WEEKS conducted a questionnaire with each mother, and community members nominated women from poor households to benefit from the cash transfer program. This process helped to ensure community buy-in for the program and transparency regarding how women were selected. For the current study, 48 communities from the larger RCT were selected within a geographically limited area to facilitate transport and set-up of the mobile neuroimaging laboratory. Infants and toddlers between 6 and 36 months old without any known neurological disorders or developmental disabilities constituted the inclusion criteria.

### 2.2 Materials

#### 2.2.1 Parent questionnaires

##### 2.2.1.1 Caregiver Reported Early Development Instruments (CREDI)

A measure of early childhood development, the CREDI asks about observable milestones and behaviors that are easily identified by caregivers.^71^ It is culturally and linguistically neutral, having been tested in 15 low-, middle-, and high-income countries. Parents were asked 20 questions to obtain a summary score for their child’s overall developmental status. For example, for 6 to 11 month old infants, questions include: “Does the child grasp onto a small object (e.g., your finger, a spoon) when put in his/her hand?,” “Can the child crawl, roll, or scoot forward on his/her own?” For children 24 to 29 months, questions include “Can the child say ten or more separate words (e.g., names like “Mama” or objects like “ball”)?, “Can the child count up to five objects (e.g., fingers, people)?” The CREDI was scored using the credi R package, which generates an overall raw scaled score and a norm-referenced standardized z-score that summarizes children’s developmental status.^72^

##### 2.2.1.2 Goldberg anxiety and depression scale (GADS)

Goldberg anxiety and depression scale (GADS) is an 18-item self-report symptom inventory^73^ measuring parents’ symptoms of anxiety and depression. Participants respond “yes” or “no” to nine items each for anxiety and depression over the last 30 days. An example includes: “Over the past month, have you felt too worried?”

##### 2.2.1.3 Household food insecurity access scale (HFIAS)

The HFIAS assesses a family’s access to food over the last month.^74^ It is an inventory of eight questions with binary “yes” or “no” answers. Six questions are followed by a Likert scale to indicate frequency. For example, “In the past four weeks, have you been worried that your household won’t have enough to eat? If yes, how many times has this happened? 1. Rarely (once or twice in the last four months), 2. Sometimes (three to ten times in the last four weeks), 3. Often (more than ten times in the last four weeks).”

##### 2.2.1.4 Multidimensional poverty index (MPI)

The MPI quantifies levels of poverty by measuring several dimensions of deprivation across households.^75^ Using a composite indicator, three dimensions of deprivation (i.e., health, education, and standard of living) were equally weighted and averaged using Alkire and Santos’ methodology.^75^ Health deprivation includes lack of food, poor assessment of health, and child mortality. Education deprivation includes having at least one child not attending school and a mother who is not educated. Living standard deprivation includes a lack of access to electricity, water, sanitation, flooring, cooking fuel, and assets such as television, iron, and a working bed. A greater MPI score equates to a greater amount of multidimensional poverty; a household is classified as multidimensionally poor if the MPI score is >0.33, and is severely poor if the MPI score is >0.55.

#### 2.2.2 fNIRS resting-state scan

**Fig. 1:**
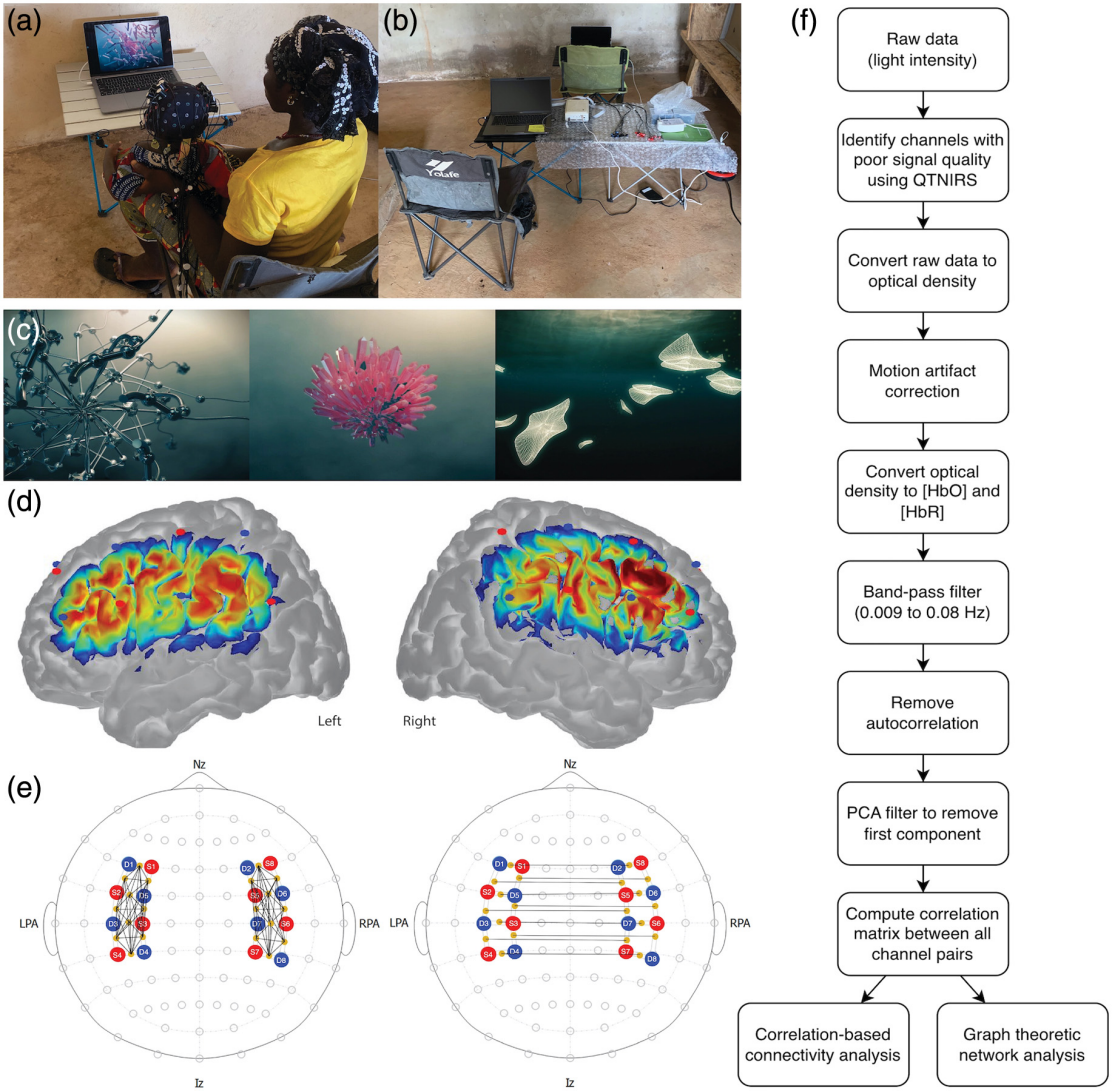
Experimental paradigm and analysis pipeline. Note. (a) Child seated in mom’s lap observing resting-state paradigm while undergoing fNIRS. (b) Testing set-up showing location of the mother and child and experimenter station in the field laboratory. (c) Example images from the inscapes resting-state paradigm. (d) Source and detector positions and sensitivity profile of fNIRS channels. (e) Source and detector positions overlaid on the 10 to 20 system; left and right images show intra- and inter-hemispheric connections in correlation analyses. (f) Data analysis pipeline.

While undergoing fNIRS neuroimaging, infants and toddlers completed a 4-min resting-state scan during which the infants and toddlers viewed the inscapes computer-generated animation designed for children to watch during neuroimaging sessions.^76^ The inscapes animation provides stimulation to engage participants while minimizing cognitive processing; the content is nonverbal and nonsocial, and no sound was presented [see Fig. 1(c)].

### 2.3 Procedure

At time 1 (2021), mothers completed a baseline interview one-on-one with a trained experimenter. At time 2 (2023 to 2024), mothers completed the CREDI questionnaire with a trained experimenter, and infants and toddlers completed a resting-state scan using fNIRS. Time 1 and time 2 visits were on average 26 months apart (M=26.21, SD=5.38, range=20 to 38). fNIRS experiment notes and CREDI data were collected and stored on REDCap.^77^

#### 2.3.1 fNIRS data acquisition

Our research employed an innovative mobile fNIRS field setup for the acquisition of neuroimaging data. This portable technology allows for research in populations that have previously been inaccessible and who reside in remote locations. All equipment is portable; experimenters traveled to remote villages and set up a mobile laboratory within schools, community buildings, or when necessary, outdoors in a laboratory tent. The lab setup required ∼30  min. To limit light, windows were closed, and/or a customized tent that blocked light was set up inside the testing space (i.e., tent was set up inside the building). Experimenters noted occasional noises (e.g., animals and vehicles) in the ambient environment, but testing space was typically set up in spaces that were quiet. Infants and toddlers were seated on their mother’s lap ∼1  m away from the monitor. The total time for a session was about 10 to 15 min, which included measurements for the fNIRS cap, cap placement, scanning, and removal of the cap. Infants and toddlers viewed 4 min of the inscapes video; this was the only task they completed. Although the original inscapes video is ∼7  min long, for infants and toddlers, the timing was shortened due to their limited attention span.

Infants’ and toddlers’ hemodynamics response was measured with a NIRx Sport NIRS system with 16 optodes (eight sources, eight detectors, yielding 20 channels) acquiring data at 10.2 Hz. The lasers were factory set to 760 and 850 nm. Before testing started, the distance between the nasion and inion (midsagittal through Cz and lateral through the preauricular point) and left to right preauricular point (through Cz) was measured and an appropriately sized flexible cap was fitted for each participant. Caps ranging from 42 to 50 cm in diameter were used to accommodate infants and toddlers with varying head sizes. Four participants wore a size 42 cm cap, 13 wore a size 46 cm cap, seven wore a size 48 cm cap, and 18 wore a size 50 cm cap. Positioning of the cap was accomplished using anatomical fiducial points (nasion, inion, left and right preauricular points). The optodes were positioned according to the 10 to 20 system in a 2×4 configuration to overlay the frontal, temporal, and parietal cortices in both the left and right hemispheres. The sensitivity profile of the fNIRS channels was generated using Monte Carlo simulation in AtlasViewer, see Fig. 1(d),^78^ and the average MNI coordinates for each channel were computed, see Table 1. Channels are visualized in Fig. 1(e).

**Table 1:** Anatomical location of fNIRS channels.

Ch.	Source	Detector	MNI coordinates			Region
			X	Y	Z	
1	1	1	−48	41	26	L. inferior frontal (pars triangularis)
2	1	5	−44	18	31	L. inferior frontal (pars opercularis)
3	2	1	−54	21	24	L. inferior frontal (pars opercularis
4	2	3	−60	1	27	L. postcentral
5	2	5	−57	14	34	L. inferior frontal (par opercularis)
6	3	3	−56	−16	39	L. supramarginal
7	3	4	−48	−24	41	L. inferior parietal
8	3	5	−44	−8	37	L. postcentral
9	4	3	−63	−29	27	L. supramarginal
10	4	4	−46	−37	32	L. supramarginal
11	5	2	54	26	43	R. middle frontal
12	5	6	57	9	30	R. precentral
13	5	7	56	−3	49	R. middle frontal
14	6	6	64	1	27	R. postcentral
15	6	7	56	−13	38	R. postcentral
16	6	8	63	−24	29	R. superior temporal
17	7	7	60	−26	57	R. inferior parietal
18	7	8	67	−40	44	R. supramarginal
19	8	2	56	39	29	R. inferior frontal (pars triangularis)
20	8	6	62	23	22	R. inferior frontal (pars opercularis)

Prior to recording, the signal-to-noise ratio was tested for each channel using NIRx Aurora software. Mothers were instructed to hold their child in their lap while the child viewed the inscapes animation on a 13-in. laptop screen. For additional details on field neuroimaging protocol and culturally relevant informed consent procedures, please see Refs. 65 and 79.

### 2.4 Analysis

#### 2.4.1 fNIRS data processing and analysis

The data analysis pipeline is visualized in Fig. 1(f). All analyses were performed in MATLAB (version 2024b) using a combination of custom functions and functions from the HOMER software (version 2).^82^

##### 2.4.1.1 Data quality

Channel quality was assessed using two measures. Channel quality was first assessed using the QT-NIRS toolbox^83^ (SCI threshold = 0.8; Q threshold=0.5, PSP threshold = 0.1) using cut-off frequencies of 1.33 to 3 Hz appropriate to infant and toddler heart rates between 80 and 180 beats per minute and 5-s nonoverlapping windows for analysis. As a secondary measure, we also calculated the signal-to-noise (SNR) ratio based on signal average and amplitude (threshold = 6) using HOMER’s enPruneChannels function. We compared QT-NIRS and SNR results to identify if the two quality metrics converged on the same number of identified channels. For three participants, QT-NIRS results were more conservative (excluded more channels) compared with SNR results; to maximize the data available for analysis, we included those channels. Participants were retained for analysis if more than 45% of channels met the specified QT-NIRS standards. Nine infants and toddlers were excluded from the study on this basis. In the retained sample, an average of 27% of channels per participant did not meet the quality thresholds in QT-NIRS and were excluded from further analysis. See the Supplementary Material for percentage of excluded channels across participants.

##### 2.4.1.2 Preprocessing

The preprocessing pipeline was developed based on the protocols of the Society for fNIRS Educational Mini-Course on Functional Connectivity with fNIRS (https://github.com/rcmesquita/rsFC-fnirs-course). Raw data were converted to optical density using HOMER’s hmrIntensity2OD function. Motion artifact correction was done with spline interpolation (spline threshold = 4.5)^84^ followed by wavelet decomposition using HOMER’s hmrMotionCorrectWavelet function with a 1.5 interquartile range to detect outliers (an IQR of 1.5 is the default in HOMER software, for a discussion of different IQR values, see Lester et al.^85^). Motion-corrected optical density values were then converted to Δ[HbO] and Δ[HbR] using HOMER’s hmrOD2Conc function with a partial pathlength factor determined by two differential pathlength factors (DPFs) accounting for two wavelengths and participants’ age.^86^ The DPFs were calculated based on the following formula: $DPF(\lambda ,A)=\alpha +\beta A^{\gamma }+\delta \lambda^{3}+\varepsilon \lambda^{2}+\zeta \lambda$ where A = age and λ = wavelength (e.g., DPF(760,1.25)=5.35, DPF(850,1.25)=4.29). Δ[HbO] and Δ[HbR] were band-pass filtered (0.009 to 0.08 Hz) using HOMER’s hmrBandpassFilt function, and autocorrelations were removed with pre-whitening.^87^^,^^88^ A PCA regression was applied to the filtered data to remove systemic physiology using HOMER’s enPCAfilter function. The first component was removed. After each processing step, data were examined for motion artifacts and visualized using HOMER’s hmrMotionArtifact function.

##### 2.4.1.3 Correlation-based connectivity analyses

**Fig. 2:**
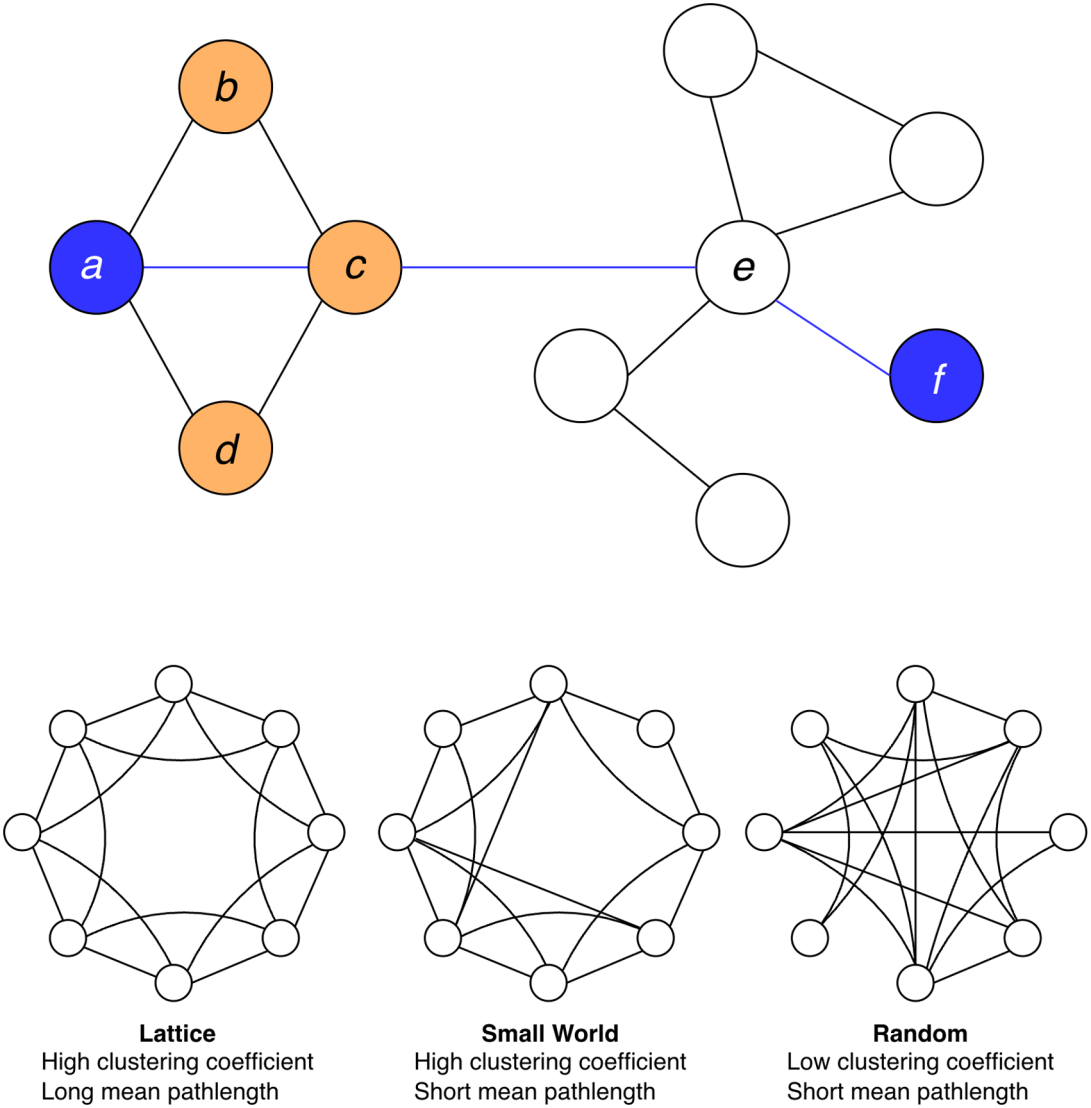
Key graph theoretic metrics. (1) Clustering coefficient: Closeness of connections between neighboring nodes. a is connected to b, c, and d. Possible pairwise connections include b–c (present), c–d (present), b–d (absent) (i.e., two out of three possible connections). Clustering coefficient is 2/3 or 0.66. (2) Local efficiency: Average shortest-path connecting all neighbors of a given node. Shortest pathlength connecting b–c, c–d, and b–d is 1, 1, and 2, respectively. Total, 4, divided by the number of neighbors, 3. Local efficiency of a is 4/3 or 1.33. (3) Degree centrality: Number of edges connected to a node. e has high degree centrality, 5. f has low degree centrality, 1. (4) Shortest pathlength: Smallest number of edges transferred from one node to another. Shortest pathlength of a to f is 3 (a–c, c–e, e–f).^80^ Lattice (regular), small world, and random networks are visualized.^81^

Correlation coefficients were calculated for HbO and HbR for all channel pairs and z-score for each participant. Intrahemispheric correlations were defined as all channel-wise pairs within each hemisphere. Interhemispheric correlations were defined as homologous channel pairs. Interhemispheric functional connectivity analyses were restricted to homologous channel pairs to capture anatomically and functionally corresponding regions across hemispheres, see Fig. 2(e).

##### 2.4.1.4 Graph theoretical network analyses

Neural systems can be modeled as networks comprised of nodes (i.e., units of the system, such as brain regions) and edges (i.e., interactions between nodes, or the functional connectivity that links nodes). Brain nodes can be defined depending on the spatial scales of interest (e.g., neuronal populations, brain regions), or imaging modality (e.g., fNIRS optodes, EEG electrodes, or groups of voxels in MRI). Edges can be defined as statistical dependencies between nodal time courses (e.g., Pearson’s correlation) typically estimated while the participant is at rest. A network with N nodes is represented by an N-by-N adjacency matrix, where nonzero elements indicate the strength of an edge between two nodes. An adjacency matrix was calculated for the HbO and HbR correlation matrix with a 0.3 threshold to binarize the correlation matrix. Nodes were defined as channels. Graph theoretical metrics were then applied to the adjacency matrix to assess the networks’ topological properties.

Graph theory metrics included (a) the clustering coefficient (the fraction of each node’s neighbors that are neighbors of each other), where each node’s clustering coefficient indicates the closeness of the connections between its neighboring nodes and the network’s clustering coefficient indicates the average clustering coefficient across all nodes in a network; a high clustering coefficient indicates high efficiency for local information transfer and is used as an indicator of network segregation;^89^ (b) local and global efficiency (a measure of interconnectedness of the entire network; global efficiency of each node is the average inverse shortest-path length from this node to all other nodes), which defines the global capacity of the network to process information via short paths and is an indicator of network integration; (c) degree centrality (or node centrality), defined as the number of edges connected to a node; nodes with high degree centrality are processing hubs with a high degree of interconnectedness and play an important role in global information transfer;^11^^,^^12^^,^^54^^,^^90^ (e) shortest pathlength (average minimum number of edges traversed between a node and all other nodes), which reflects the network’s capability for global information integration; a shorter path length indicates higher efficiency in global information transfer; and (f) small-world architecture;^91^ see Fig. 2. Small-world networks are characterized by a combination of a high clustering coefficient and short pathlength. Networks with high clustering coefficient and long pathlength are classified as regular, and networks with low clustering coefficient and short pathlength are classified as random.^89^ The small-worldness of a network can be quantified by comparing the clustering coefficient and path length in a real network to a random network.^91^^–^^93^

#### 2.4.2 Brain-behavior analyses

Separate linear regression models were calculated for each neural network measure with age, gender, CREDI score, perinatal maternal mental health, and perinatal MPI as predictors using the lm function in R.^94^ FDR correction was applied by calculating q-values, calculated across all nine primary neural outcomes and all predictors combined, using the *qvalue* (fdr=0.05) function from the Storey’s q-value estimator.^95^ Correlations between variables were tabulated using *apaTables*,^96^ and visualizations were generated using *ggplot*.^97^ Treatment status was not significantly associated with any variables and was therefore not included in final models. We also conducted linear regression models for each neural network measure with age, gender, CREDI score, and concurrent maternal mental health and MPI as predictors, reported in the Supplementary Material.

## 3 Results

### 3.1 Behavioral Results

Descriptive behavioral results are displayed in Table 2. Children with higher CREDI scores were statistically significantly more likely to have educated mothers. Higher MPI was statistically significantly associated with poorer maternal overall health and mental health, higher food insecurity, less maternal education, and less household assets, see Table 2. Poor maternal mental health was also significantly associated with maternal health, child mortality, food insecurity, and household assets. All correlations are displayed in Table 3.

**Table 2:** Descriptive statistics.

Variable	N	Mean	SD	Min	Max
Infant and toddler age (months)	42	15.1	7.66	6	36
6 to 11 months	13	30.95%			
12 to 17 months	17	40.48%			
18 to 36 months	12	28.57%			
Gender	42				
Male	20	47.60%			
Female	22	52.40%			
Maternal age	42	32.2	7.18	21	55
CREDI score	42	0.000911	0.966	−1.56	2.01
Maternal mental health (higher score = more symptoms)	42	0.717	0.247	0.167	1
Multidimensional poverty index (higher score = greater poverty)	42	0.479	0.162	0.17	0.86
Maternal health (1 to 5; 1 = excellent, 5 = poor)	42	3.45	0.832	1	5
Child mortality	42	47.6%			
Food insecurity (# of meals/day in last week)	42	2.45	0.55	1	3
Household food insecurity scale	42				
None	1	2.4%			
Mild	3	7.1%			
Moderate	14	33.3%			
Severe	24	57.1%			
Maternal education (mother attended school)	42	42.9%			
School-aged children in household enrolled in school	42	78.8%			
Electricity	42				
National electricity network	18	42.9%			
Generator	12	28.6%			
Flashlight	7	16.7%			
Flashlight from mobile phone	2	4.8%			
Other electricity distributor	1	3%			
Sanitation	42				
No toilet installed/in bush or field	20	47.6%			
Pit toilet/latrine	17	40.5%			
Open pit	4	9.5%			
Neighbor’s toilet	1	2.4%			
Flooring	42				
Cement	31	73.8%			
Clay	9	21.4%			
Hardened dung	2	4.8%			
Water access (time in minutes to fetch water)	42	19.2	33.5	1	180
Cooking fuel	42				
Firewood	39	92.9%			
Charcoal	3	7.1%			
Assets (% of households that do not own an iron, mobile phone, fan, bed, radio, tv, or motorbike)	42	35.7%			

**Table 3:** *Correlations between variables*.

Variable	1	2	3	4	5	6	7	8	9	10
1. Child age										
2. Maternal age	−0.11									
	[−0.40, 0.20]									
3. CREDI	−0.19	0.08								
	[−0.47, 0.12]	[−0.23, 0.37]								
4. MPI (higher score = more poverty)	−0.25	0.13	0.18							
	[−0.51, 0.06]	[−0.18, 0.41]	[−0.13, 0.46]							
5. Maternal mental health (higher score = more symptoms)	−0.33*	0.37*	0.12	0.51**						
	[−0.57, −0.03]	[0.08, 0.61]	[−0.19, 0.41]	[0.24, 0.70]						
6. Maternal health	−0.20	−0.26	0.17	0.43**	0.45**					
	[−0.47, 0.11]	[−0.52, 0.05]	[−0.14, 0.45]	[0.14, 0.65]	[0.17, 0.66]					
7. Child mortality	−0.24	0.32*	0.18	0.15	0.28	0.06				
	[−0.50, 0.07]	[0.01, 0.57]	[−0.13, 0.46]	[−0.16, 0.44]	[−0.02, 0.54]	[−0.25, 0.35]				
8. Maternal education	−0.14	0.11	−0.17	−0.40**	0.21	0.05	−0.15			
	[−0.42, 0.18]	[−0.20, 0.40]	[−0.45, 0.14]	[−0.63, −0.11]	[−0.11, 0.48]	[−0.26, 0.35]	[−0.44, 0.16]			
9. Child School Enrollment (% of children in household enrolled in school)	−0.48**	0.26	0.31*	−0.09	0.23	0.15	0.55**	0.12		
	[−0.68, −0.21]	[−0.05, 0.52]	[0.00, 0.56]	[−0.38, 0.22]	[−0.08, 0.50]	[−0.16, 0.43]	[0.29, 0.73]	[−0.19, 0.41]		
10. Assets (higher score = less assets)	−0.30	0.11	−0.01	0.56**	0.39*	0.44**	−0.21	0.16	−0.01	
		[−0.56, 0.00]	[−0.20, 0.40]	[−0.31, 0.29]	[0.30, 0.74]	[0.10, 0.62]	[0.15, 0.65]	[−0.49, 0.10]	[−0.15, 0.44]	[−0.31, 0.29]	
11. Food Insecurity (higher score = more food insecure)	−0.30	0.29	0.16	0.39*	0.77**	0.38*	0.04	0.26	0.17	0.48**	
	[−0.56, 0.00]	[−0.01, 0.55]	[−0.15, 0.44]	[0.10, 0.62]	[0.61, 0.87]	[0.08, 0.61]	[−0.27, 0.34]	[−0.05, 0.52]	[−0.14, 0.45]	[0.21, 0.69]	

Values in square brackets indicate the 95% confidence interval for each correlation.

* indicates p<.05.

** indicates p<.01.

**Table 4:** *Predictors of network metrics and functional connectivity (HbO)*.

Predictor	Cluster coefficient (segregation)			Global efficiency (integration)			Degree centrality (integration)			Shortest pathlength (integration)			Small worldness			Intra-hemi. Corr.			L. intra-hemi. corr.			R. intra-hemi. corr.			Inter-hemi. corr.		
	β(SE)	p	q	β(SE)	p	q	β(SE)	p	q	β(SE)	p	q	β(SE)	p	q	β(SE)	p	q	β(SE)	p	q	β(SE)	p	q	β(SE)	p	q
Age	**0.395** **(0.154)**	0.014*	0.022*	**0.579** **(0.146)**	< 0.001***	< 0.001***	**0.532** **(0.149)**	0.001**	0.005**	0.103 (0.163)	0.531	0.145	**−0.457** **(0.16)**	0.007**	0.013*	**0.495** **(0.15)**	0.002**	0.006**	**0.479** **(0.154)**	0.004**	0.010*	0.166 (0.174)	0.346	0.116	0.198 (0.175)	0.268	0.104
Gender (ref=F)	0.264 (0.296)	0.379	0.116	0.134 (0.282)	0.638	0.154	0.394 (0.288)	0.18	0.081	−0.435 (0.321)	0.185	0.081	−0.123 (0.309)	0.694	0.162	0.273 (0.29)	0.352	0.116	−0.139(0.296)	0.641	0.154	0.538 (0.336)	0.118	0.067	0.119 (0.335)	0.724	0.164
CREDI	**0.329** **(0.152)**	0.037*	0.039*	**0.227** **(0.145)**	**0.126**	**0.067**	0.166 (0.148)	0.267	0.104	0.086 (0.169)	0.615	0.154	**−0.324** **(0.158)**	0.049*	0.045*	0.134 (0.149)	0.372	0.116	0.118 (0.152)	0.442	0.128	0.059 (0.172)	0.735	0.164	0.002 (0.173)	0.991	0.211
MMH	**0.339** **(0.172)**	**0.057**	0.046*	**0.33** **(0.164)**	**0.052**	0.045*	**0.249** **(0.167)**	**0.145**	**0.073**	**0.317** **(0.184)**	**0.095**	**0.057**		0.487	0.137	0.188 (0.168)	0.272	0.104	0.185 (0.172)	0.288	0.106	0.059 (0.195)	0.763	0.166	0.1 (0.194)	0.61	0.154
MPI	**−0.377** **(0.171)**	0.034*	0.039*	**−0.284** **(0.163)**	**0.089**	**0.057**	**-0.305** **(0.166)**	**0.074**	**0.055**	0.246 (0.179)	0.179	0.081	0.106 (0.178)	0.555	0.148	**−0.362** **(0.167)**	0.037*	0.039*	**−0.307** **(0.171)**	**0.08**	**0.055**	−0.169 (0.193)	0.387	0.116	−0.183 (0.193)	0.349	0.116
$R^{2}$	0.299			0.363			0.34			0.216			0.24			0.329			0.3			0.1			0.069		

MMH, maternal mental health (higher = more symptoms); MPI, multidimensional poverty index (higher = more poverty). Variance inflation factors for all variables in all models were below 2, which indicates no collinearity in the models.^98^ ^†^ indicates p<.1, * indicates p<.05, ** indicates p<.01, *** indicates p<.001. Bolded text indicates significant and marginally significant effects. HbR models are shown in the Supplementary Material.

### 3.2 Brain Results

**Fig. 3:**
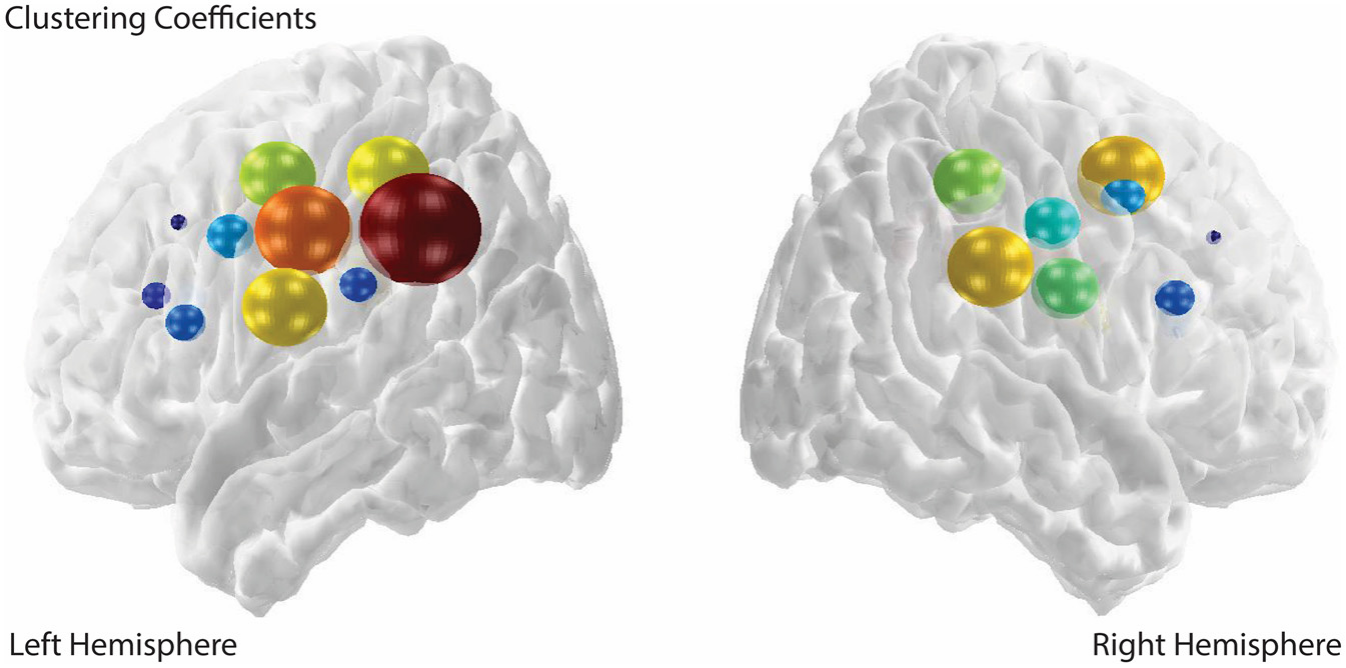
Average clustering coefficients in each brain region.

Overall, children demonstrated small-world network architecture, as indicated by a small-worldness index over 1 (M=3.71; SD=2.59), and high clustering coefficients (M=0.34, SD=0.15) accompanied by short pathlength (M=2.09, SD=0.50). Clustering coefficients were larger in posterior regions than anterior regions, see Fig. 3. Clustering coefficients for channel 10 (left-most posterior) were significantly higher than channel 1 (left-most anterior; t(34)=3, p=.003). Similarly, clustering coefficients for channel 18 (right-most posterior) were significantly higher than channel 19 (right-most anterior; t(34)=4, p<.001).

### 3.3 Brain-Behavioral Results

**Fig. 4:**
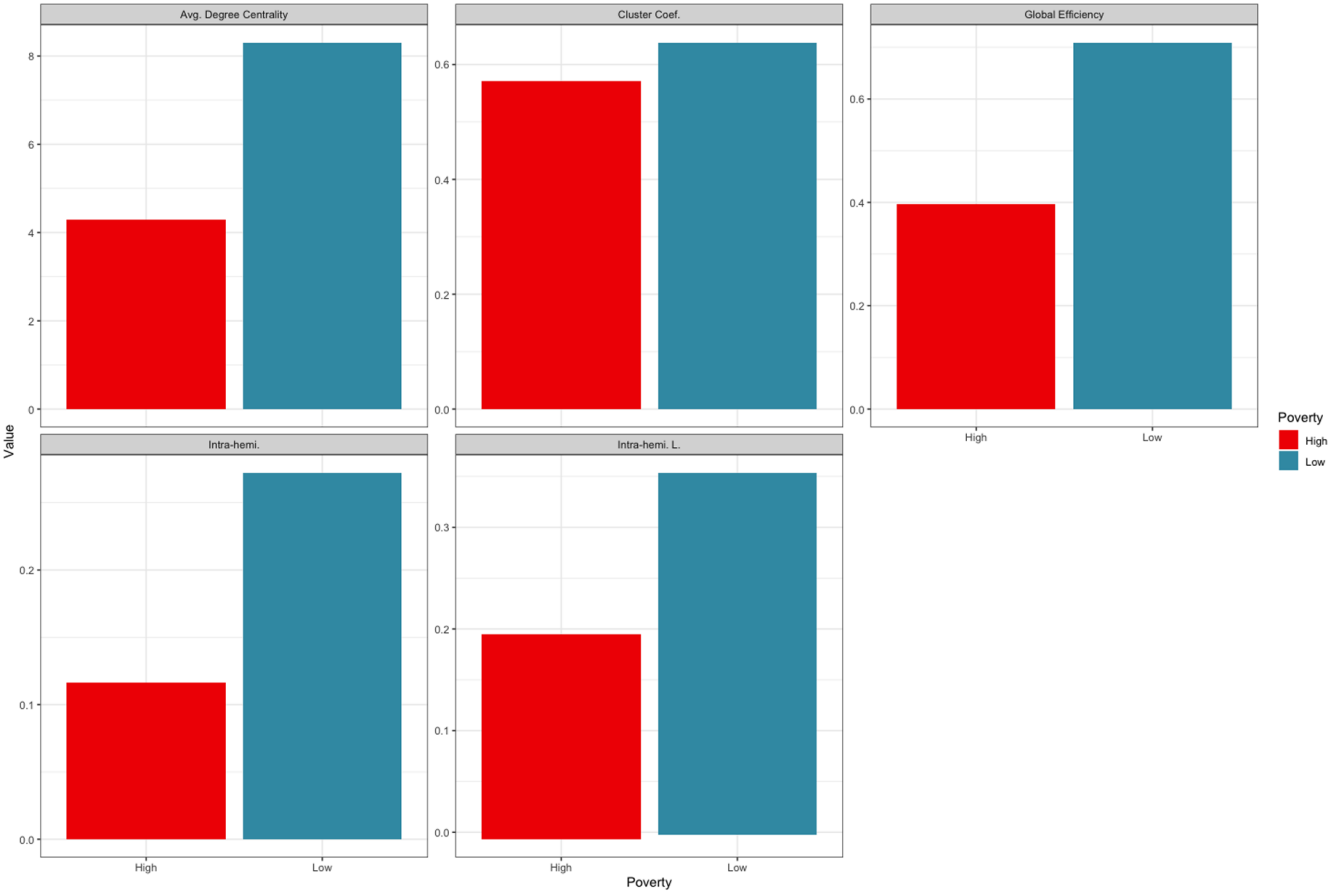
Network metrics across high and low poverty levels. High poverty level corresponds to MPI scores in excess of one standard deviation above the mean MPI scores. Low poverty level corresponds to MPI scores less than one standard deviation below the mean MPI scores. Only network metrics with significant or marginal effects of poverty are shown.

Models for all network metrics are displayed in Table 4. Overall, higher MPI was statistically significantly associated with lower clustering coefficient and intra-hemispheric correlation and marginally associated with lower global efficiency and degree centrality. Poorer maternal mental health was associated with greater clustering coefficient and global efficiency and marginally associated with greater degree centrality and shortest pathlength. Older children showed higher clustering coefficients, global efficiency, degree centrality, and intra-hemispheric correlation, specifically left intra-hemispheric correlation, but lower small-worldness. Higher CREDI scores were associated with lower small-worldness, but greater clustering coefficients and marginally greater global efficiency. No statistically significant associations were observed between gender and any network metrics. Average network metrics for participants with high (more than one standard deviation above the mean MPI score) and low (less than one standard deviation below the mean MPI score) MPI scores are shown in Fig. 4.

## 4 Discussion

We investigated the relation between early-life environments and neural network development 2 years later in infants and toddlers from economically vulnerable households in rural Côte d’Ivoire. We measured child development and neural network organization using fNIRS, a portable and noninvasive neuroimaging method. By analyzing functional connectivity and network properties, we aimed to examine how greater poverty (measured by multidimensional deprivation) and stress exposure (measured by maternal mental health symptoms) around the time of conception and in utero were linked to neural network integration and segregation and functional connectivity 2 years later. We observed associations of both poverty and stress with infant and toddler neural connectivity and network organization. Overall, more developed (i.e., older, higher CREDI scores) children showed greater integration and segregation, but lower small-worldness. Exposure to poverty was negatively associated with segregation, integration, and functional connectivity. Conversely, poor maternal mental health was positively associated with segregation and integration. These effects suggest that deprivation and exposure to stress may be differentially linked to early-life neural networks.

### 4.1 Developmental Patterns in Neural Network Organization

Consistent with existing research,^99^^,^^100^ we found evidence that small-world architectures were already present in infants and toddlers in our sample, indicated by small-worldness indices above one,^91^ high clustering coefficients, and short pathlengths. Older children showed overall greater segregation and integration and left intra-hemispheric correlation, but lower small-worldness. Similarly, greater maturation (indicated by higher developmental assessment scores) was also associated with higher segregation and lower small-worldness. We found no gender differences in network architecture, which is consistent with previous reports showing gender differences in children emerge after 2 years of age.^57^

Although overall integration and segregation increase over development, the developmental trajectories for neural networks show nonlinear patterns in the first 3 years of life. For example, Nazari and Salehi found an overall increase in global and local efficiency, clustering coefficients, and small worldness over development, albeit with nonlinear u-shaped patterns.^100^ Similarly, Tymofiyeva et al.^56^ found that small worldness increased in the first 6 months of life and then decreased until adulthood. Indeed, regressive events play an important role in modifying neural connectivity early in development.^101^ For example, synaptic density peaks around 2 years of age and subsequently declines by 50% to 60% in adulthood.^102^ The postnatal period is characterized by ongoing synaptogenesis (this process peaks at 34 weeks gestational age when ∼40,000 new synapses are formed per second,^103^ and pruning of excess synaptic connections, largely occurring in the first 2 years of life. These combined progressive (i.e., synaptogenesis) and regressive (i.e., synaptic pruning) processes support the development and refinement of neural networks.^13^^,^^104^ These developmental trajectories align with age-related changes in graph theoretic metrics.^105^^,^^106^ For example, Hassett et al.’s study of functional connectivity suggests that developmental changes in network density (the number of connections compared with all possible connections in a network) parallel the reduction and refinement of synapses in childhood.^105^ Similarly, Tomasi and Volkow^107^ suggest that age-related changes in clustering, path length, and small-worldness are consistent with maturation-related pruning. The observed negative association between age and developmental assessment scores with small-worldness in the present study may similarly reflect the regressive processes of brain development in the first 3 years of life.

### 4.2 Poverty, Maternal Mental Health, and Neural Network Organization

Recent advances in developmental science reflect a shift from broad cumulative risk approaches toward dimensional models that aim to clarify how a vast range of early-life adversities are associated with developmental outcomes via specific mechanisms.^24^^,^^25^^,^^108^^–^^110^ Two influential frameworks have emerged in this regard. The Dimensional Model of Adversity and Psychopathology^24^^,^^25^ proposes that early-life adversities (including growing up in a family with mental health problems) can be organized along two key dimensions: threat, defined as experiences involving harm or the threat of harm, and deprivation, defined as the absence of expected environmental inputs or cognitive and social stimulation. In parallel, the life history model characterizes early-life adversity along the dimensions of harshness—exposure to extrinsic dangers such as violence or mortality—and unpredictability, or inconsistent and chaotic environmental conditions.^111^

Although the experiences of early-life adversity are vast, the current study investigated two common, and often co-occurring adversities that can involve high degrees of both deprivation and threat: poverty and low maternal mental health. Poverty is often characterized by deprivations in nutrition and reductions in cognitively stimulating activities. Although distinct from threat, it often co-occurs with environmental threats, dangers, and harms such as community violence. Poor maternal mental health has also been linked to contextual threats and unpredictability, including increased stress reactivity, interpersonal violence, and poorer parent–child interactions, as well as biological threats such as increased cortisol reaction to stress.^38^^,^^39^^,^^112^

We build on these frameworks to capture the complexity of children’s early environments, identifying three core dimensions: neglect/deprivation (conceptualized here using MPI as a proxy), threat/harm, and unpredictability (conceptualized here using maternal mental health as a proxy).^113^ These distinctions offer a more mechanistic model of how experiences of adversity may become biologically embedded and may differentially influence neurodevelopment. Exposure to adversity, including deprivation, threat, and unpredictability, has been linked to changes in cortical structure, functional connectivity, and network organization, with implications for cognitive abilities and emotional responses.^114^^–^^118^

#### 4.2.1 Deprivation and neural network organization

In line with fNIRS findings from Bangladesh and The Gambia,^49^^,^^51^^,^^52^^,^^119^ we find that poverty is related to neurodevelopment in the context of rural Côte d’Ivoire. Poverty-related links to network organization are likely in part due to the diminished nutritional intake of mothers and infants exposed to poverty. Food insecurity is highly correlated with malnutrition, an established pathway linking poverty and neurocognitive development.^31^^,^^32^ Indeed, in our sample, food insecurity rates were very high, with 80.4% categorized as moderately or severely food insecure. For example, Turesky et al.^50^ identified relations between malnutrition and brain structure in infants in a high-poverty sample in Bangladesh.

Related literature has found increased small-worldness and lower global efficiency in preterm infants relative to full-term infants, which the authors interpreted as an indicator of accelerated development conferring resilience against prematurity-related pathology.^120^ Zhao et al.^121^ found that diminished parental care can affect functional neural development in children; they found that left-behind children (i.e., children who are left behind when their parents migrate for economic or political reasons) show decreased network efficiency. Increased small-worldness is also associated with exposure to adversity in adults. For example, post-deployment veterans with post-traumatic stress disorder (PTSD) showed higher small-worldness and clustering coefficient compared with participants without current PTSD.^122^ Early adversity also impacts the organization of the structural connectome in mice by altering neural wiring.^123^ Research using network modeling has shown that these adverse conditions increase the randomness of neural connections while simultaneously reducing the efficiency of long-distance wiring.^123^ This shift in organizational parameters suggests that early-life adversity may lead to less optimized structural connectivity in the brain, potentially impairing cognitive functions later in development.^123^^,^^124^ However, the changes observed in network organization could represent an adaptive mechanism to future environmental challenges, highlighting the complex interplay between stress and neural development.^123^

#### 4.2.2 Threat and unpredictability, and neural network organization

Recent work shows that elevated maternal anxiety symptoms are related to higher maternal unpredictability signals.^43^^,^^44^^,^^125^ Emotionally predictable caregiving has long been linked to neurodevelopment and behavioral outcomes [e.g., Refs. 126, 127]. Indeed, rodent models show that maternal unpredictability from the dam to the pup resulted in alterations to the neural circuits related to cognitive and emotional development in the pup.^128^^,^^129^ Deprivation simulations in rodent models (i.e., restricting nesting materials) result in fragmented and unsystemic caregiving behavior in the rat dam, which consequently led to anhedonia and memory impairment in pups.^128^^,^^129^

Importantly, neurodevelopment is not only a product of one’s current environment, but rather one’s environment over the course of development. There is a large body of existing literature highlighting relations between prenatal maternal mental health and infant neurodevelopment, as well as those between prenatal socioeconomic status and infant neurodevelopment [e.g., Refs. 130, 131], that supports the importance of in-utero development. A few studies have directly compared prenatal versus postnatal symptoms (or model both) and find timing-specific associations with brain outcomes. For example, in a South African cohort, antenatal and postpartum depressive symptoms predicted child brain structure around age 2 to 3 years.^132^ Concurrent maternal mental health and poverty at age 2 might miss earlier sensitive windows—i.e., pregnancy effects can be stronger because they reflect direct neurodevelopmental embedding.^133^^,^^134^ In exploratory analyses, concurrent MPI and MMH were not significantly associated with neural outcomes (see the Supplementary Material); however, these null results should be interpreted cautiously given limited power and intervening environmental changes that were not captured in the study.

Our observation that deprivation (related to poverty) and stress associated with threat and unpredictability (related to poor maternal mental health) were differentially related to neural network organization suggests that deprivation and stress may be distinctly related to differences in developing brains. In our sample, infants and toddlers whose mothers reported poorer perinatal maternal mental health showed higher integration and segregation, but those with higher exposure to poverty showed lower segregation, integration, and functional connectivity. Recent research suggests that adversity in early life can result in accelerated brain development, particularly in regions relevant to emotional processing and cognitive control.^135^^,^^115^ For example, leveraging the Philadelphia Neurodevelopmental Cohort, Gur et al.^114^ found that exposure to poverty (low SES) and exposure to traumatic stressful events were associated with earlier onset of puberty, an indicator of accelerated maturation.

The positive relation between stress (related to poor maternal mental health) and integration and segregation observed in the present study may be an indicator that exposure to threat and unpredictability relates to accelerated development, albeit our small sample size and narrow age range of 6 to 36 months limit drawing strong conclusions. Importantly, studies utilizing a developmental framework highlight that resilient children show adaptive brain changes, potentially leading to enhanced social and cognitive skills despite early stress.^136^^,^^137^ Although neurodevelopment results from complex and dynamic interactions between genomic and environmental processes, recent theoretical advances highlight the role of stochastic processes as an intrinsic element of brain organization.^138^ Stochasticity, which refers to the probabilistic nature and intrinsic unpredictability of development, heightens intraindividual variability and adaptive features that support success in challenging environments. In a series of computational models simulating the effect of stochasticity on brain organization, Carozza et al.^138^ showed that unpredictability is associated with more resilient networks (i.e., robust to perturbation and less susceptible to temporary increase in noise). The authors show that brain networks in children from low SES backgrounds (environments characterized by greater unpredictability) are well simulated with more stochastic models. Carozza et al.^138^ advanced the adaptive stochasticity hypothesis, whereby heightened stochasticity within the developing brain may serve as an adaptive mechanism in situations of environmental uncertainty. Indeed, our findings that increased exposure to threat and unpredicability—more unpredictable and less sensitive caregiving linked to poor maternal mental health—is related to greater integration and segregation align with the adaptive stochasticity hypothesis.

### 4.3 Limitations and Future Directions

We explored theoretical mechanisms of deprivation, threat, and unpredictability to unpack the link between early-life adversity and neurobiology; however, it is critical to acknowledge that these theories manifest from research in the Minority World. Increased research in Majority World, rural contexts—where early-life adversities are much more prevalent and abundant, e.g., Refs. 139–141—will likely yield updated theoretical explanations for the links between adversity and development. Although the adaptive stochasticity hypothesis is aligned with our neurobiological results, we do not know whether our observed results will relate to increased resilience later in development. Furthermore, little longitudinal work has been done to date to understand the long-term impacts of stochastic processes. Although these findings propose a paradigm shift toward understanding adversity as a catalyst for certain adaptive developmental processes,^137^^,^^142^^,^^143^ we emphasize the need for longitudinal research, especially in Majority World settings, to understand the neurobiological deficits and adaptations that occur in the face of early-life adversity.

The link between poverty and neural network organization observed here also suggests that neurodevelopmental patterns may be due to diminished nutritional intake of both mothers and infants. Indeed, we observed a significant link between food insecurity and poverty, indicating that mothers experiencing greater poverty are likely missing key nutrients necessary for brain development, in the preconception period, in utero, and postnatally [i.e., via breastfeeding; 144]. Animal studies show that maternal undernutrition during gestation contributes to alterations in glial markers, neuronal processes, and neurotrophic factors, an increased apoptosis ratio, and disrupted gene expression patterns,^145^ which in turn impacts the downregulation of signaling pathways crucial for neurogenesis and axon guidance for synaptogenesis. Remarkably, maternal undernutrition during preconception also significantly impacts brain development; for example, a brief period of protein restriction preconception was sufficient to impair fetal cortical development and hippocampal-dependent recognition memory in rat offspring.^146^ Maternal undernutrition also leads to intrauterine growth restriction,^147^ which is associated with altered network organization and negative neurodevelopmental outcomes.^148^ An important future direction would be to examine direct links between malnutrition, including anthropometric data, and neurodevelopment.

An important limitation of this work is the combination of a small sample size and heterogeneity of timing, both with respect to pregnancy and age of participants. Participants in our sample consist of infants and toddlers between 6 and 36 months of age; however, given the small sample size, we are not able to stratify participants by heterogeneity in maternal pregnancy stage at time 1 nor by participant age at time 2. Previous research shows that maternal mental health and poverty may differentially relate to neurocognitive development based on children’s developmental stage.^49^ Moreover, maternal mental health profiles change across pregnancy and postpartum stages.^149^ In particular, although we did not find significant associations between concurrent maternal mental health and neural outcomes in our exploratory analyses, several factors may have contributed to the null result, including limited study power, narrow age range of 6 to 36 months, and variation in measurement timing, as well as intervening factors that were not measured. Nonetheless, we believe this study constitutes an important contribution to the field, highlighting relations between maternal factors and neurocognitive development in a Majority World context where families experience high levels of adversity. However, future work should investigate whether and how maternal factors differentially relate to neurocognitive development based on developmental period and pregnancy timing in Majority World contexts, including testing potential mediation pathways between dimensions of deprivation, threat, and unpredictability, neurocognitive development, and child outcomes.

Last, it is important to note several methodological limitations of the present study. Chiefly, the sample size is small, limiting additional analyses that could deepen the findings. Variation in preprocessing and analysis parameters may also contribute to results (or lack thereof), including quality thresholds that retain more data but may introduce noise. Infant and toddler data are notoriously challenging to collect and prone to noise (e.g., due to fussiness, motion artifacts), which has contributed to the fNIRS toddler “data desert,” e.g., Ref. 150. Future work should seek to replicate these results and include underrepresented populations such as the sample in this study. Given these limitations, we remain cautious in our interpretation and highlight the need for additional replication work.

### 4.4 Implications for Children’s Development

A growing body of evidence indicates that effects of poverty and stress on neurobiological development are likely central to poverty-related disparities in academic achievement. Disadvantaged environments characterized by economic insecurity, nutritional deficiencies, and poor stimulation (disproportionately overrepresented in the Majority World) lead to brains adapted to such environments, and in turn, put children at a disadvantage for the development of basic cognitive skills that support academic success.^32^^,^^151^^,^^152^ Income-based disparities in brain development persist over childhood and into adulthood. For example, in a cross-sectional sample of 389 children aged 4 to 22 years, children from impoverished families showed reduced gray matter volume in frontal and temporal cortices, and the hippocampus.^153^ Moreover, disrupted early brain development results in delayed motor skills, which may in turn interfere with exploration associated with cognitive development. Furthermore, a child’s short stature (i.e., associated with malnutrition) may reduce expectations from parents and peers,^29^ further compounding the negative effects of impoverished environments on the brain.

Our longitudinal findings extend this literature by showing that poverty and poor maternal mental health during the prenatal period and early life are linked to differences in neural network organization in the first 3 years of life. These findings underscore the importance of structural poverty reduction efforts—including policies that increase household income, reduce food insecurity, and improve access to quality healthcare and nutrition before and during pregnancy. Such interventions have the potential to buffer against the biological embedding of adversity and promote healthy brain development during this critical period. Indeed, a related randomized controlled trial of the impacts of cash transfers to economically vulnerable mothers in rural Côte d’Ivoire (mothers in the treatment condition received 8€ each week for 100 weeks) showed significant improvements in four of six indicators of economic well-being (d=0.23 to 0.75) and reductions in maternal stress (d=−0.27).^154^

At the same time, our results highlight the need for interventions that support maternal well-being. Poor maternal mental health is associated with altered caregiving behaviors and greater environmental unpredictability,^44^^,^^155^^,^^156^ both of which were linked in this study to differences in infants’ neural network integration and segregation. Provision for psychosocial supports and programs that enhance caregiver sensitivity and stability could help mitigate the impact of early adversity on brain development. Because adversity-related differences in neural network organization may precede observable behavioral problems, investing in maternal mental health during the perinatal period represents a highly strategic opportunity for prevention.

## 5 Conclusion

The period of rapid developmental change in the first 3 years of life corresponds to a period of heightened neural plasticity during which the effects of adverse experiences, including poverty-related material deprivation and stress associated with poor maternal mental health, can dramatically affect neurocognitive development. This study examined how early-life environments characterized by poverty and poor maternal mental health relate to neural network development during the first few years of life. Using portable fNIRS, we found that greater poverty exposure was associated with lower network segregation and integration, whereas poorer maternal mental health was linked to greater segregation and integration. These findings suggest that deprivation and stress may differentially influence early neural network organization and could represent distinct pathways through which adversity affects neurodevelopment. Taken together, the results highlight the importance of addressing the structural drivers of poverty that limit access to essential resources and undermine maternal mental health and, in turn, caregiving quality. Poverty reduction interventions for young families and interventions to improve maternal mental health have the potential to maximize protective effects during the prenatal and early postnatal periods, when neural circuits are most malleable. Furthermore, leveraging portable neuroimaging methods such as fNIRS to identify early biomarkers of risk may help refine the timing and targeting of interventions in low-resource settings.

## Supplementary Material

10.1117/1.NPh.13.S1.S13014.s01

## Data Availability

Data and scripts are available at https://osf.io/9yn84/.
