# Supplementary material for: Poverty and caregiver mental health are related to infant and toddler resting-state brain networks: insights from fNIRS
Source: Neurophotonics. 2026 Aug 14;13(Suppl 1):S13014. doi: 10.1117/1.NPh.13.S1.S13014 (PMC13473268; doi:10.1117/1.NPh.13.S1.S13014)
Supplement: Supplementary file 1 [file NPh_013_S13014_SD001.pdf]

Supplementary Table 1

Concurrent Predictors of Network Metrics and Functional Connectivity (HbO)

Predictor	Cluster Coefficient (segregation)		Global Efficiency (integration)		Degree Centrality (integration)		Shortest Pathlength (integration)		Small worldness		Intra-hemi. Corr.		L. Intra-hemi. Corr.		R. Intra-hemi. Corr.		Inter-hemi. Corr.	
	β(SE)	*p*	*β(SE)*	*p*	*β(SE)*	*p*	*β(SE)*	*p*	*β(SE)*	*p*	*β(SE)*	*p*	*β(SE)*	*p*	*β(SE)*	*p*	*β(SE)*	*p*
Age	**0.398 (0.173)**	**.028***	**0.559 (0.162)**	**.001****	**0.572 (0.163)**	**.001****	-0.18 (0.185)	.339	**-0.43 (0.168)**	**.015***	**0.558 (0.166)**	**.002****	**0.435 (0.167)**	**.013***	**0.305 (0.178)**	**.096** †	0.3 (0.181)	.106
Gender (ref=F)	0.239 (0.312)	.449	0.168 (0.292)	.57	0.364 (0.293)	.222	-0.224 (0.332)	.504	-0.159 (0.303)	.603	0.18 (0.299)	.551	-0.173 (0.3)	.569	0.444 (0.321)	.175	0.001 (0.326)	.998
CREDI (higher = more dev.)	**0.296 (0.174)**	**.097** †	0.193 (0.163)	.243	0.15 (0.163)	.363	0.089 (0.189)	.64	**-0.287 (0.169)**	**.097** †	0.111 (0.167)	.511	0.037 (0.167)	.828	0.115 (0.179)	.523	0.074 (0.179)	.684
MMH (higher = worse symptoms)	-0.085 (0.178)	.637	-0.077 (0.167)	.647	-0.146 (0.167)	.389	**0.361 (0.191)**	**.067** †	-0.007 (0.173)	.969	-0.125 (0.171)	.469	0.106 (0.171)	.542	-0.293 (0.183)	.119	-0.194 (0.186)	.303
MPI (higher = more poverty)	0.084 (0.168)	.618	0.121 (0.157)	.448	0.099 (0.158)	.534	-0.121 (0.172)	.489	-0.091 (0.163)	.579	0.068 (0.161)	.677	0.07 (0.162)	.668	0.018 (0.173)	.917	-0.14 (0.177)	.433
R^2	0.188		0.288		0.285		0.126		0.234		0.254		0.25		0.143		0.102	

Note. MMH = Maternal Mental Health, MPI = multidimensional poverty index. Variance inflation factors for all variables in all models was below 2, which indicates no collinearity in the models [97]. † indicates $p < .1$, * indicates $p < .05$, ** indicates $p < .01$, *** indicates $p < .001$.

Supplementary Table 2

Concurrent Predictors of Network Metrics and Functional Connectivity (HbR)

Predictor	Cluster Coefficient (segregation)		Global Efficiency (integration)		Degree Centrality (integration)		Shortest Pathlength (integration)		Small worldness		Intra-hemi. Corr.		L. Intra-hemi. Corr.		R. Intra-hemi. Corr.		Inter-hemi. Corr.	
	β(SE)	*p*	*β(SE)*	*p*	*β(SE)*	*p*	*β(SE)*	*p*	*β(SE)*	*p*	*β(SE)*	*p*	*β(SE)*	*p*	*β(SE)*	*p*	*β(SE)*	*p*
Age	-0.061 (0.186)	.744	0.28 (0.184)	.137	0.146 (0.188)	.442	0.257 (0.187)	.18	-0.194 (0.18)	.288	0.089 (0.19)	.644	0.089 (0.181)	.627	0.026 (0.183)	.887	-0.039 (0.181)	.839
Gender (ref=F)	-0.107 (0.334)	.751	-0.081 (0.331)	.807	0.048 (0.338)	.887	0.181 (0.336)	.594	-0.355 (0.324)	.28	-0.083 (0.342)	.809	-0.251 (0.327)	.448	0.193 (0.329)	.561	0.214 (0.338)	.531
CREDI (higher = more dev.)	0.26 (0.186)	.171	0.224 (0.184)	.232	0.226 (0.184)	.232	-0.196 (0.191)	.313	-0.154 (0.18)	.4	0.144 (0.19)	.455	0.202 (0.182)	.273	-0.033 (0.183)	.859	-0.022 (.186)	.905
MMH (higher = worse symptoms)	0.051 (0.191)	.793	-0.106 (0.189)	.579	-0.081 (0.193)	.677	-0.105 (0.193)	.59	-0.049 (0.185)	.791	-0.097 (0.195)	.623	0.082 (0.187)	.665	-0.262 (0.188)	.171	-0.082 (0.193)	.675
MPI (higher = more poverty)	-0.036 (0.18)	.84	0.052 (0.178)	.771	0.033 (0.182)	.859	0.099 (0.175)	.575	-0.181 (0.174)	.305	0.052 (0.184)	.778	0.125 (0.176)	.48	-0.08 (0.177)	.654	-0.049 (0.183)	.791
R^2	0.071		0.088		0.05		0.104		0.126		0.025		0.111		0.1		0.032	

Note. MMH = Maternal Mental Health, MPI = multidimensional poverty index. Variance inflation factors for all variables in all models was below 2, which indicates no collinearity in the models [97]. † indicates $p < .1$, * indicates $p < .05$, ** indicates $p < .01$, *** indicates $p < .001$.

Supplementary Table 3

Predictors of Network Metrics and Functional Connectivity (HbR)

Predictor	Cluster Coefficient (segregation)		Global Efficiency (integration)		Degree Centrality (integration)		Shortest Pathlength (integration)		Small worldness		Intra-hemi. Corr.		L. Intra-hemi. Corr.		R. Intra-hemi. Corr.		Inter-hemi. Corr.	
	β(SE)	*p*	*β(SE)*	*p*	*β(SE)*	*p*	*β(SE)*	*p*	*β(SE)*	*p*	*β(SE)*	*p*	*β(SE)*	*p*	*β(SE)*	*p*	*β(SE)*	*p*
Age	-0.011 (0.176)	.95	0.284 (0.174)	.111	0.117 (0.179)	.518	0.284 (0.168)	.1	-0.24 (0.175)	.178	0.061 (0.182)	.741	0.133 (0.175)	.454	-0.076 (0.18)	.675	-0.085 (0.179)	.636
Gender (ref=F)	-0.127 (0.339)	.71	-0.14 (0.335)	.679	0.038 (0.346)	.912	0.066 (0.331)	.843	-0.256 (0.337)	.452	-0.115 (0.35)	.744	-0.313 (0.338)	.36	0.223 (0.347)	.524	0.239 (0.342)	.49
CREDI	0.265 (0.174)	.137	0.206 (0.172)	.24	0.211 (0.178)	.244	-0.199 (0.175)	.263	-0.216 (0.173)	.219	0.125 (0.18)	.491	0.261 (0.173)	.14	-0.14 (0.178)	.436	-0.067 (0.176)	.706
MMH	0.159 (0.197)	.423	0.183 (0.195)	.353	-0.007 (0.201)	.972	0.328 (0.19)	.093.	-0.006 (0.196)	.974	-0.012 (0.203)	.953	-0.018 (0.196)	.927	0.004 (0.201)	.985	-0.081 (0.198)	.686
MPI	-0.08 (0.195)	.682	-0.049 (0.193)	.803	0.023 (0.199)	.908	-0.116 (0.185)	.536	-0.038 (0.194)	.848	0.061 (0.202)	.766	0.019 (0.195)	.922	0.073 (0.2)	.719	0.081 (0.197)	.685
R^2	.299		.363		.34		.216		.24		.329		.3		.1		.069	

Note. MMH = Maternal Mental Health (higher = worse symptoms), MPI = multidimensional poverty index (higher = more poverty). Variance inflation factors for all variables in all models was below 2, which indicates no collinearity in the models [100]. † indicates *p* < .1, * indicates *p* < .05, ** indicates *p* < .01, *** indicates *p* < .001.

Supplementary Table 4

Percentage of Excluded fNIRS Channels

Ch.	% of participants with low quality data
1	38
2	36
3	38
4	21
5	33
6	7
7	14
8	14
9	7
10	2
11	45
12	24
13	21
14	29
15	10
16	12
17	12
18	10
19	52
20	36
